# The effects of peppermint on exercise performance

**DOI:** 10.1186/1550-2783-10-15

**Published:** 2013-03-21

**Authors:** Abbas Meamarbashi, Ali Rajabi

**Affiliations:** 1 Department of Physical Education and Sports Sciences, University of Mohaghegh Ardabili, Ardabil 56199-11367, Iran

**Keywords:** Peppermint essential oil, Exercise performance, Respiratory gas analysis, Spirometry

## Abstract

**Background:**

Enhancing athletic performance is a great desire among the athletes, coaches and researchers. Mint is one of the most famous natural herbs used for its analgesic, anti-inflammatory, antispasmodic, antioxidant, and vasoconstrictor effects. Even though inhaling mint aroma in athletes has been investigated, there were no significant effects on the exercise performance.

**Methods:**

Twelve healthy male students every day consumed one 500 ml bottle of mineral water, containing 0.05 ml peppermint essential oil for ten days. Blood pressure, heart rate, and spirometry parameters including forced vital capacity (FVC), peak expiratory flow rate (PEF), and peak inspiratory flow (PIF) were determined one day before, and after the supplementation period. Participants underwent a treadmill-based exercise test with metabolic gas analysis and ventilation measurement using the Bruce protocol.

**Results:**

The FVC (4.57 ± 0.90 vs. 4.79 ± 0.84; p < 0.001), PEF (8.50 ± 0.94 vs. 8.87 ± 0.92; p < 0.01), and PIF (5.71 ± 1.16 vs. 6.58 ±1.08; p < 0.005) significantly changed after ten days of supplementation. Exercise performance evaluated by time to exhaustion (664.5 ± 114.2 vs. 830.2 ± 129.8 s), work (78.34 ±32.84 vs. 118.7 ± 47.38 KJ), and power (114.3 ± 24.24 vs. 139.4 ± 27.80 KW) significantly increased (p < 0.001). In addition, the results of respiratory gas analysis exhibited significant differences in VO_2_ (2.74 ± 0.40 vs. 3.03 ± 0.351 L/min; p < 0.001), and VCO_2_ (3.08 ± 0.47 vs. 3.73 ± 0.518 L/min; p < 0.001).

**Conclusions:**

The results of the experiment support the effectiveness of peppermint essential oil on the exercise performance, gas analysis, spirometry parameters, blood pressure, and respiratory rate in the young male students. Relaxation of bronchial smooth muscles, increase in the ventilation and brain oxygen concentration, and decrease in the blood lactate level are the most plausible explanations.

## Background

Until now, many researches have been done on the effectiveness of various kinds of natural products in the improvement of sport performances. Mint (mentha) is a herb which is well known for its antispasmodic, painkilling [1-3], anti-inflammatory, antispasmodic, decongestant, and antioxidant effects [4]. Peppermint is one of the mentha species (*i.e.*, mentha piperita, peppermint oil, mentha arvensis, cornmint oil) [5]. Menthol (29%) and menthone (20-30%) are the major components of the peppermint essential oil.

External application of peppermint extract raised the pain threshold in human [6]. Peppermint aroma was also effective on perceived physical workload, temporal workload, effort, and anxiety [7]. Another research demonstrated the effectiveness of peppermint aroma administered through the nose or by mouth on the augmenting cognitive performance [8]. Peppermint aroma caused improvement on the tasks related to attentional processes, virtual recognition memory, working memory, and visual-motor response [8]. Moreover, peppermint aroma improved the typing performance [9]. In a study under four conditions (peppermint, jasmine, dimethyl sulfide, or a non-odorous), athletes performed a 15-minute treadmill exercise stress test, then mood and exercise performance were evaluated [10]. Perceived physical workload, temporal workload, and self-evaluated performance reported to have a significant difference in peppermint group. In an animal study, intraperitoneal injection of different components of peppermint into mice, significantly increased the ambulatory activity. Therefore, author suggested peppermint components are serving as a central nervous system stimulant [11].

The effect of supplementation with oral peppermint extract was also studied on the perceived lower leg muscular pain and blood lactate levels one hour before a 400-m running test [12]. In this study, the peppermint had a significant effect on the blood lactate level, but not on the muscle pain. Besides, the combination of peppermint oil and ethanol [13] reported to have a significant analgesic effect.

Using a Peak Flow Meter device showed an improvement in the lung capacity and inhalation ability after inhalation of peppermint aroma [14]. After inhalation of peppermint aroma, the nasal airflow force increased, thus the author speculated this effect supply more oxygen to the brain, which could be effective for continuing physical performance. On the other hand, menthol the main component of the peppermint essential oil investigated in a four-week randomised, placebo-controlled study on 23 patients with chronic asthma. Menthol group shown no significant differences in the vital capacity, forced expiratory volume or change in the peak expiratory flow rate [15]. Moreover, previous study on the athletic performance by using peppermint essential oil had no significant effect on the blood oxygen saturation, pulse rate, blood pressure, and mean arterial pressure (MAP) [16].

The possible ergogenic effect of aromas, has certainly received much publicity in recent years. However, there is very little scientific evidence to support or refute the claims made by merchants, practitioners, and manufacturers [17]. Hence, due to equivocal findings and lack of good-quality evidences on the effectiveness of peppermint essential oil in the exercise performance, the aim of this study was to assess the effects of oral supplementation with peppermint essential oil on the exercise performance, physiological and respiratory parameters.

## Methods

### Subjects and study design

Twelve (12) healthy male university students (M_age_ = 25.9 ± 1.38 yrs; M_weight_ = 69.9 ± 5.58 kg; M_height_ = 177.0 ± 4.2 cm) randomly selected among 40 volunteers to take part in a quasi experiment by using the one-group pre-test, post-test design. Participants tested before, and after ten days of peppermint essential oil consumption. Ethical approval to conduct this study obtained from the University Human Ethics Committee.

### Methodology

All participants signed a consent form. The subjects were familiarized with the laboratory setting and the measurement techniques two days before the study. Blood pressure, breath rate, and resting heart rate were recorded. The chest circumference was measured by placing the flexible measuring tape around the chest at the level of the xipho-sternal junction. Pulmonary function tests performed using a handheld electronic turbine spirometer (Microlab spirometer, Micro Medical Limited of Rochester, England) and the best of three forced efforts such as forced vital capacity (FVC), peak expiratory flow rate (PEF), and peak inspiratory flow (PIF) were recorded.

Finally, participants underwent a standard treadmill exercise test (Bruce protocol), controlled by a computer program. A heart rate transmitter belt (Polar, Polar Electro, Finland) was attached to the chest to transmit the heart rate signals to the receiver. Respiratory gas and ventilation were measured with calibrated PowerCube Gas Analyzer (Ganshorn Medizin Electronic GmbH, Nie derlauer, Germany). Gas exchange variables including: oxygen uptake (L/min), carbon dioxide production (L/min), ventilation (L/min), breathing rate (min^-1^), respiratory gas-exchange ratios, and other parameters recorded every ten seconds. Exercise performance parameters consist of time to exhaustion (TE), total work (W_total_), maximal power (P_max_), vertical distance, and horizontal distance computed by the treadmill’s software considering the slope angle, speed and duration of each stage.

Each participant consumed one bottle of mineral water (500 ml) per day, containing 0.05 ml peppermint essential oil for ten days. All the tests repeated after ten days of supplementation. Participants were asked to refrain from any medium to vigorous exercise and their diet was controlled during the study.

### Statistical analyses

Normal distribution was tested using the Kolmogorov-Smirnov and Shapiro-Wilk tests. Paired t-test used to examine differences between pre-test and post-test. To calculate the magnitude of the difference between pre-test and post-test, a Cohen’s d calculated, using the following formula [18]:

$$\text{Choen's d}=\frac{m_{1}-m_{2}}{\sqrt{\frac{\left(n_{1}-1\right)SD_{1}^{2}+\left(n_{2}-1\right)SD_{2}^{2}}{\left(n_{1}+n_{2}+2\right)}}}$$

Cohen’s d of 0.20 considered a minor, 0.50 a medium, and 0.80 a major difference. The statistical analysis performed using the Statistical Package for Social Sciences software (SPSS Version 16, SPSS Inc. Chicago, IL).

## Results

After ten days of supplementation with peppermint essential oil, the exercise performance evaluated by changes in the physiological parameters (spirometry and gas analysis) and functional indicators of exercise performance. The Kolmogorov-Smirnov and Shapiro-Wilks tests revealed the normality of the data. The parameters obtained from the gas analyzer during Bruce test presented in the Table 1.

**Table 1:** Physiological parameters obtained by gas analyser in Pre-test and Post-test

**Parameter**	**Pre-test (n = 12)**	**Post-test (n = 12)**	**Changes%**	**T**	**P value**	**Effect size**
**VO**_**2 **_**[L/min]**	2.74 ± 0.40	3.03 ± 0.351	10.5	6.757	p < 0.001	0.775
**VCO**_**2 **_**[L/min]**	3.08 ± 0.47	3.73 ± 0.518	21.1	5.594	p < 0.001	1.319
**VE [L/min]**	84.60 ± 17.74	116.80 ± 22.44	38	4.790	p < 0.001	1.592
**RR**	39.26 ± 9.24	50.53 ± 7.33	28.7	5.683	p < 0.001	1.352
**PETO**_**2 **_**[mmHg]**	88.87 ± 4.19	96.25 ± 4.02	8.3	5.869	p < 0.001	1.798
**PETCO**_**2 **_**[mmHg]**	40.86 ± 4.28	35.16 ± 3.78	−16.2	7.270	p < 0.001	1.412
**DFCO**_**2**_**/DFO**_**2**_	1.109 ± 0.053	1.233 ± 0.072	7.4	4.233	p < 0.005	1.962
**RER**	1.147 ± 0.052	1.247 ± 0.066	8.7	3.873	p < 0.005	1.690
**VO**_**2**_**/Kg [ml/kg/min]**	39.25 ± 3.69	43.63 ± 3.78	11.1	5.912	p < 0.001	1.174
**VCO**_**2**_**/Kg [ml/kg/min]**	44.95 ± 4.61	54.29 ± 6.45	20.7	4.769	p < 0.005	1.666
**VE/Kg [ml/kg/min]**	1229.9 ± 212.13	1692.6 ± 296.5	37.6	4.306	p < 0.005	1.795
**EQO**_**2**_	30.60 ± 4.65	38.80 ± 4.13	26.7	4.984	p < 0.001	1.865
**EQCO**_**2**_	26.20 ± 3.65	31.20 ± 2.78	19	6.578	p < 0.001	1.542
**VT [L]**	2.165 ± 0.489	2.536 ± 0.404	17.1	6.770	p < 0.001	0.827
**VA [L]**	86.00 ± 19.22	117.31 ± 22.22	36.4	4.492	p < 0.005	1.507
**METS**	11.21 ± 1.06	12.48 ± 1.07	11.3	6.054	p < 0.001	1.192
**EE [kcal/h]**	847.60 ± 123.64	955.10 ± 116.98	12.6	6.138	p < 0.001	0.893
**FETO**_**2 **_**[%]**	14.95 ± 0.70	16.35 ± 0.55	9.3	6.917	p < 0.001	2.232
**FETCO**_**2 **_**[%]**	6.681 ± 0.679	5.800 ± 0.507	−15.1	6.102	p < 0.001	1.470
**CHO [kcal/h]**	1276.7 ± 232.39	1721.4 ± 327.85	34.8	4.170	p < 0.005	1.565
**FAT [kcal/h]**	323.38 ± 124.04	691.06 ± 223.77	13.6	4.834	p < 0.001	2.032

Data are expressed as mean ± SD.

Functional parameters significantly improved in post-test as compared with pre-test. A substantial increase in the respiratory ventilation, respiratory rate (RR), VO_2_/Kg, VCO_2_/Kg, MET, and energy expenditure were observed showing enhancement in the respiratory efficiency and energy expenditure during the exercise. An increase in the breathing rate, normally leads to a lower alveolar and arterial PCO_2_ and therefore, decrease in the end-tidal carbon dioxide tension (PETCO_2_) and fractional end-tidal CO_2_ concentration (FETCO_2_) expected (Table 1).

Time to exhaustion, vertical distance, horizontal distance, maximum work, and power compared and presented in the Table 2.

**Table 2:** Changes in the exercise performance parameters

**Parameter**	**Pre-test (n = 12)**	**Post-test (n = 12)**	**Changes%**	**T**	**P value**	**Effect size**
**Horizontal distance (m)**	843.5 ± 234.6	1187.6 ± 309.2	40.7	6.890	p < 0.001	1.254
**Vertical distance (m)**	113.4 ± 40.09	172.8 ± 59.41	52.3	6.262	p < 0.001	1.173
**Work (KJ)**	78.34 ± 32.84	118.7 ± 47.38	51.5	5.746	p < 0.001	0.992
**Power (KW)**	114.3 ± 24.24	139.4 ± 27.80	21.9	6.764	p < 0.001	0.962
**Time to exhaustion (S)**	664.5 ± 114.2	830.2 ± 129.8	24.9	7.255	p < 0.001	1.355

Data are expressed as mean ± SD.

Functional indicators of exercise performance showed significant increase in the time to exhaustion and distance (Table 2). In the Tables 3 and 4, the lung function indicators and other physiological parameters compared between pre-test and post-test.

**Table 3:** Spirometry parameters in the Pre-test and Post-test

**Parameter**	**Pre-test (n = 12)**	**Post-test (n = 12)**	**Changes%**	**T**	**P value**	**Effect size**
**FVC (L)**	4.57 ± 0.90	4.79 ± 0.84	4.8	6.336	p < 0.001	0.258
**PEF (L/s)**	8.50 ± 0.94	8.87 ± 0.92	4.35	3.446	p < 0.01	0.401
**PIF (L/s)**	5.71 ± 1.16	6.58 ± 1.08	15.1	4.505	p < 0.005	0.776

Data are expressed as mean ± SD.

**Table 4:** Cardiopulmonary parameters obtained from the Pre-test and Post-test

**Parameter**	**Pre-test (n = 12)**	**Post-test (n = 12)**	**Changes%**	**T**	**P value**	**Effect size**
**Resting heart rate**	65.18 ± 12.72	62.18 ± 11.82	−4.8	3.609	p < 0.005	0.244
**Maximum heart rate**	173.4 ± 14.35	187.4 ± 15.17	8	3.777	p < 0.005	0.954
**Systolic blood pressure**	11.99 ± 0.87	11.28 ± 0.85	−6.2	5.440	p < 0.001	0.824
**Diastolic blood pressure**	6.645 ± 0.503	6.164 ± 0.566	−7.8	7.831	p < 0.001	0.900
**Chest circumference at max. inhale**	89.41 ± 4.59	89.95 ± 4.66	0.6	2.782	p < 0.05	0.118
**Chest circumference at max. exhale**	83.73 ± 5.28	82.41 ± 5.14	−1.6	4.342	p < 0.005	0.253

Data are expressed as mean ± SD.

Lung function tests significantly increased after ten days of supplementation. Peak inspiratory flow (PIF) shows maximum changes whereas forced vital capacity (FVC) had least changes and effect size.

Both resting and exercise heart rates were significantly decreased during post-test. Similarly, the chest circumference during maximum exhale and blood pressure in the post-test significantly decreased.

## Discussion

Previous studies have shown that various kinds of mint were effective in reducing muscle pain [19,20], muscle relaxation, and reduce fatigue [21]. However, previous studies showed inhaling peppermint aroma has no effect on the lung function tests and physical performance during acute and intensive exercise [18]. In a research on the effect of peppermint aroma during 15-minute low intensity treadmill exercise among male and female college students [22], no significant difference seen in the resting or exercise heart rate, oxygen consumption, ventilation, and perceived physical workload.

In the current research, improvement in the spirometric measurements (FVC, PEF, and PIF) and ventilation during treadmill exercise, as well as an increase in the maximum chest circumferences observed. These results can be justified by decreasing the airway and bronchial smooth muscle tonicity with or without effect on the pulmonary surfactant. Previously, reported a significant increase in the respiratory muscle strength after four-week inspiratory and expiratory muscle training on the respiratory muscle strength and time to exhaustion in healthy people [15]. In the current study, PIF, which is dependent on strength and speed of shortening of the inspiratory muscles, significantly improved. Therefore, an increase in the inspiratory muscle strength after peppermint consumption is conceivable.

In an in-vitro study, menthol vapour lowered the surface tension on synthetic surfactant films [23]. It may change the lung surface tension and its function [23]. Bronchodilatory effect of peppermint is unlikely because previous research [24] investigated the effect of salbutamol as a β_2_-adrenergic receptor agonist and with bronchodilator effect on the cycling performance. However, there was no significant difference in any variables related to aerobic endurance or cycling performance [24]. In yet another four-week randomised placebo controlled study, 23 subjects with chronic mild asthma received either nebulised menthol (10 mg twice a day) or placebo. No effect on the forced expiratory volume reported in the experimental group. However, the menthol group significantly decreased their bronchodilator medicines and had fewer wheezing episodes [15]. It can be speculated that oral supplementation in the current study is preferred to longer time nebulised menthol administration. We suggest further investigations on the hepatic metabolism of the peppermint essential oil components to elucidate the pharmacokinetics of peppermint absorbed through the nose, mouth or intestine.

The result of the current study supports the theory that delaying fatigue may be related to physiological changes by decreasing blood lactate level similar to the recent finding [25]. Furthermore, significant increase in the carbohydrate metabolism after ten days of supplementation (Table 1) is implying that peppermint can improve the muscular energy metabolism. Further studies are needed to elucidate the possible effects of peppermint in the cellular energy metabolism.

The stimulating effect of peppermint on the CNS [11] may also be responsible. Extensive research on the effectiveness of aromas on cognitive performance, perceived physical workload, and pain responses were conducted based on possible changes in the brain activity [3,7,16,18,22,26-28].

Table 1 demonstrated significant changes in the gas analysis results after ten days of supplementation with peppermint essential oil. In the supplementation phase, subjects kept their physical activity in minimum level, therefore; plausible explanation would be a positive effect of supplementation on the cardiovascular and respiratory efficiency. Positive changes in carbohydrate and fat oxidation in accordance with enhancement of energy expenditure and MET may be related to some unknown effects on the cellular level. Although reported that peppermint may accentuate energy by stimulating the adrenal cortex [29], it is unclear what dosage and how this increased energy may affect the exercise performance. In other studies [22,28], aroma had no significant effects on the oxygen consumption in both low-intensity 15-minute treadmill task and sub-maximal treadmill running test.

It seems peppermint has a lowering effect on the heart rate and the systolic blood pressure. Reduction in the arterial smooth muscle tonicity is a possible explanation for these effects. One study administered peppermint aroma by nose and failed to find any significant effect in both heart rate and blood pressure. The differences in the peppermint essential oil method of administration and supplementation period, can justify the effectiveness of peppermint in the current study.

## Conclusions

To our knowledge, this is the first study that explored the effect of oral supplementation with peppermint essential oil on the exercise performance. Our results strongly support the effectiveness of peppermint essential oil on the exercise performance, respiratory function variables, systolic blood pressure, heart rate, and respiratory gas exchange parameters. Differences in duration of study and oral supplementation instead of inhalation of peppermint aroma could be the important characteristics of this study compare to the previous researches. Further investigations are required to unravel the mechanism underlying the effectiveness of peppermint on the exercise performance and respiratory parameters.

## Abbreviations

FVC: Forced vital capacity; PEF: Peak expiratory flow rate; PIF: Peak inspiratory flow; Wtotal: Calculated total work; Pmax: Maximal power output; CNS: Central nervous system; VO2: Oxygen uptake; VCO2: Carbon dioxide elimination; VE: Minute ventilation; RR: Respiratory rate; PETO2: End-tidal oxygen tension; PETCO2: End-tidal carbon dioxide tension; RER: Respiratory exchange ratio; EQO2: Ventilatory equivalents for O_2_; EQCO2: Ventilatory equivalents for CO_2_; VT: Tidal volume; VA: Alveolar ventilation; METS: Metabolic equivalent; EE: Energy expenditure; FETO2: Fractional end-tidal O_2_ concentration; FETCO2: Fractional end-tidal CO_2_ concentration; CHO: Carbohydrate.

## Competing interests

Authors of this paper have not received any financial remuneration for preparing this paper. The authors declare that they have no competing interests.

## Authors’ contributions

The authors’ responsibilities were as follows–A.M. is responsible for research design, conducting laboratory tests, statistical analysis and manuscript preparation. A.R. was responsible for subject recruitment and laboratory tests assistance. Both authors read and approved the final manuscript.

## Authors’ information

Dr. Abbas Meamarbashi is Associate Professor and Head of the Department of Physical Education and Sport Science at the University of Mohaghegh Ardabili. He has been published in many peer-reviewed journals. Sport nutrition is one of his fields of interest. Mr. Ali Rajabi is an MSc student in sport physiology.
